# Supplementary material for: Knowledge, perception, and attitude toward premarital screening among university students in Kurdistan region–Iraq
Source: PLOS Glob Public Health. 2024 Nov 18;4(11):e0003515. doi: 10.1371/journal.pgph.0003515 (PMC11573136; doi:10.1371/journal.pgph.0003515)
Supplement: Attachment — Submitted filename: Response reviewers.docx [file pgph.0003515.s002.docx]

Many thanks dear Johanna Pruller and dear reviewers here are my answers regarding the comments. Again I appreciate your help to enrich our manuscript.

Dear reviewer 1: Many thanks for your comments. There are my comments.

Reviewer #1: Abstract :
Based on a preliminary review, the abstract appears well-structured and informative. It clearly outlines the study's objectives, methodology, key findings, and implications.

Answer: many thanks dear reviewer for your kind comment.

Introduction :
Clearly states the significance of PMS programs for public health
Emphasizes the need for university students to be informed about PMS
Mentions the limitations in current knowledge about student awareness of PMS
Links the study to the broader goal of improving future generations' well-being

Here are some suggestions for improvement:
• Briefly mention the specific research questions or hypotheses that the study aims to address.
• Consider providing a stronger justification for why university students were chosen as the target population for this study.

Answer: all the suggestions were considered and fixed the issues.

Method
This section provides a comprehensive overview of the methodological approach. However, some details could be further elaborated on in the full manuscript:
Regarding Questionnaire and Scoring System
1. Could authors please provide the specific questions used to assess participants' knowledge of premarital screening? It would be beneficial to include these questions in an appendix for reference.
2. Could authors elaborate on the development of the scoring system used to categorize participants' knowledge, perception, and attitude as poor, fair, or good? What specific criteria were used to determine these cut-off points?

Answer: the questionnaire was online survey as mentioned in the manuscript, and thanks again for your comment I put as an appendix and attached file. As well as the calculation was done based on the previous study in the same area while in the different country.

**Result**

Combining tables in the Results section can improve readability and reduce redundancy. Explore using data visualization techniques like charts or graphs to represent complex data trends more effectively than tables.

Given that Table 3 provides a summary of key parameters, consider re incorporating the detailed data from Tables 4 and 5 into this on sequential display. This will improve the flow of the results section and make it easier for readers to understand the key findings.

Table 6 and Table 7 can be simplified. What is the basis for determining the age of 20 and 22? I think it is not very relevant for the age limit to be displayed as one of the parameters. Class level is more meaningful.

While it's important to summarize key findings from tables in the text, avoid simply restating the data verbatim. Focus on interpreting the results and explaining their significance. Consider using descriptive statistics (means, standard deviations, percentages) and effect sizes to convey the data concisely and effectively. This will help to improve the readability of the results section and strengthen the overall impact of your findings.

The Results section should focus on presenting the most critical findings that directly address the research questions. Avoid overwhelming readers with excessive statistical detail. Instead, prioritize key results and use tables or figures to supplement the text.

Answer: Regarding the tables, I have changed table 3 into graphs since it is easy to understand, as well as I reviewed all the tables and changed some of them. Regarding the age classification it is low in Iraq and Kurdistan region students go to university at age 18, generally, as well as we classified age, in general, dividing age into two-year intervals allows for a more thorough and precise understanding of how university students' attitudes and behaviors are influenced by their age.

As well as class level or age is the same because in each level of the study they have same age for example stage 1 is 18, generellay stage 1 they have similar age, and so on.

Over all I revised the result section many thanks for your generous comments.

Discussion

The Discussion section provides a good overview of the study's findings in relation to existing literature. To enhance its impact, consider the following suggestions:

Strengthen the Argument and Interpretation
• Deepen the Interpretation: Explore the underlying reasons for the observed differences in knowledge, perception, and attitude between groups (e.g., gender, marital status, residential area). Consider potential sociocultural, economic, or educational factors influencing these disparities.
• Theoretical Framework: If applicable, integrate a theoretical framework to provide a more structured interpretation of the findings. For instance, a theory of planned behavior or health belief model could help explain the relationships between knowledge, perception, and attitude.
• Comparative Analysis: Expand the comparison with previous studies to include more recent and relevant research. Highlight the unique contributions of your study to the existing body of knowledge.

do not repeat result section into discussion (like in the first paragraph).

Why do your findings align with those of other studies conducted in the same region?
• What are the potential underlying factors contributing to this consistency?
• How can you provide a more in-depth analysis to explain these similarities beyond simply stating the concurrence?

You mention : “The findings pertaining to gender differences in knowledge may differ, and this could be explained by socioeconomic and cultural factors. For example, because they are the focus of distinct education programs, women are traditionally better educated about PMS and marriage-related issues”. Please add more reasonable finding to strengten the remark. You can compare with another region on similar finding and please adjust the potential reasoning, add <https://doi.org/10.37268/mjphm/vol.20/no.3/art.407>

While the studies cited demonstrate a generally positive perception of PMS, further research is needed to explore the nuances of these attitudes across different cultural and socioeconomic contexts.

The high level of support for PMS among participants underscores the importance of investing in public education campaigns to increase awareness and uptake of these services.

It would be beneficial to compare the findings of this study with those from other regions to identify global trends in attitudes towards premarital screening.

Overall comment for discussion section: To enhance the depth and impact of your findings, consider providing more in-depth explanations and interpretations for each statement on each paragraphs. This will help to reveal the underlying significance of the results and contribute to a more comprehensive understanding of the research topic.

Answer:

Many thanks for your comment I have reviewed the discussion section and added as you mentioned.

Reviewer #2: The manuscript meets the basic requirements for publication in PLOS Global Public Health, with technically sound research and data that supports the conclusions. However, I recommend minor revisions to address the following points:
- there are minor issues such as the lack of discussion on the reliability and validity of the questionnaire and there is a minor inconsistency in the reported sample size. Initially, it mentions a sample size of 500, but the study ended with 960 respondents. Clarifying this discrepancy and explaining the larger-than-expected response rate would be beneficial.
- Improve the manuscript’s clarity and readability through grammatical corrections, simplified sentence structures, and consistent terminology.

Answer: many thanks for your kind comments.

Regarding the questionnaire previously other researches used same questions that why we didn’t mention validity and reliability, and we mentioned in method section.

In terms of sample size, it is true the real sample size was 500, while during the survey online 960 persons answered the questions.

Regarding the grammar we checked, again many thanks for your comments.
